# Biomimetic Silk Fibroin Scaffolds Functionalized with Hydroxyapatite and Platelet Growth Factors for Bone Tissue Engineering

**DOI:** 10.3390/biomimetics10100703

**Published:** 2025-10-17

**Authors:** Mauro Pollini, Carmen Lanzillotti, Maria Antonietta De Sangro, Maria Rosaria Cazzato, Luciano Abbruzzese, Federica Paladini

**Affiliations:** 1 Department of Experimental Medicine, University of Salento, Via Monteroni, 73100 Lecce, Italy; federica.paladini@unisalento.it; 2 Caresilk S.r.l.s., Via Monteroni c/o Technological District DHITECH, 73100 Lecce, Italy; 3 Servizio di Immunoematologia e Medicina Trasfusionale, Ospedale Vito Fazzi, 73100 Lecce, Italy

**Keywords:** silk fibroin, hydroxyapatite, platelet lysate, platelet growth factors, bone tissue engineering, hemopoietic stem cells, biomimetics

## Abstract

Non-union fractures represent a significant clinical challenge requiring innovative therapeutic approaches. Silk fibroin (SF) scaffolds have gained recognition as advantageous biomaterials for bone tissue engineering due to their biocompatibility and mechanical characteristics. This study investigated the biocompatibility and osteoinductive potential of SF scaffolds functionalized with hydroxyapatite (HA) and loaded with platelet growth factors (PGFs) using hematopoietic stem cells (HSCs). SF scaffolds were prepared and functionalized with HA through methanol impregnation, while PGFs were obtained from platelet lysate via apheresis procedures. HSCs were cultured on different experimental groups, namely SF, SF-HA, PGF, SF-PGF, and SF-HA-PGF, assessing biocompatibility through 3-(4,5-dimethylthiazol-2-yl)-2,5-diphenyltetrazolium bromide (MTT) assay, Live/Dead staining, and cytoskeleton analysis over 7 days. Osteoinductive properties were evaluated using Alizarin Red staining for mineral matrix deposition at 14 and 21 days. The MTT assay revealed the biocompatibility of all the experimental groups. The Live/Dead assay confirmed high cell viability, while the cytoskeleton analysis revealed well-organized actin filaments comparable to controls. Alizarin Red staining showed that PGF alone promoted early mineral matrix deposition at day 14, while SF-HA, SF-PGF, and SF-HA-PGF groups demonstrated significantly enhanced mineralization at day 21 compared with SF alone. The combination of silk fibroin scaffolds with platelet growth factors alone or with hydroxyapatite and platelet growth factors creates a biomimetic environment that supports cell viability and induces the osteogenic differentiation of hemopoietic stem cells. These findings suggest significant potential for clinical translation in treating non-union fractures and bone defects.

## 1. Introduction

Bone tissue possesses a remarkable intrinsic regenerative capacity, with the ability to heal fractures through a complex cascade of cellular and molecular events involving inflammation, repair, and remodeling phases [1]. However, this natural healing process fails in approximately 5–10% of all fractures, leading to delayed union or non-union complications that represent a significant clinical challenge in orthopedic surgery [2]. Non-union fractures are defined as fractures that fail to heal within 9 months or show no radiographic evidence of healing progression for 3 consecutive months [3]. These complications affect over 790,000 patients annually in the United States alone, imposing substantial healthcare costs exceeding USD 500 million and causing prolonged patient morbidity, functional disability, and reduced quality of life [4,5]. The pathophysiology of impaired bone healing involves multiple interconnected factors, including inadequate vascularization, insufficient mechanical stability, compromised cellular activity, and deficient growth factor availability at the fracture site [6,7]. Traditional treatment approaches, such as autologous bone grafting, remain the gold standard but are associated with significant limitations including donor site morbidity, restricted graft availability, and hypothetical complications at harvest sites [8,9].

Bone tissue engineering represents a potential therapeutic solution to overcome these clinical challenges by combining fundamental elements such as scaffolds for providing structural support and guiding tissue formation, cells capable of osteogenic differentiation, and bioactive molecules that stimulate regenerative processes [10].

Silk fibroin (SF), derived primarily from Bombyx mori cocoons, has gained considerable attention as a biomaterial for bone tissue engineering treatments due to its unique combination of features [11]. This natural protein polymer exhibits excellent biocompatibility, controllable biodegradation rates, remarkable mechanical strength comparable to natural bone collagen, and the ability to support osteoblast adhesion and proliferation [12]. Silk fibroin’s β-sheet crystal structure provides mechanical stability, while its amorphous regions allow for flexibility and cell–material interactions. Furthermore, silk fibroin can be processed into various forms including films, fibers, sponges, and three-dimensional scaffolds using aqueous-based methods that avoid harsh organic solvents, making it particularly suitable for incorporating bioactive compounds [13].

The enhancement of silk fibroin scaffolds through functionalization with bioactive components represents a rational approach for improved performances [14]. Hydroxyapatite (HA), the primary inorganic component of natural bone, has been extensively investigated as a bioactive ceramic for bone applications due to its osteoconductivity and ability to promote calcium phosphate precipitation [15]. When combined with silk fibroin matrices, HA can provide nucleation sites for new bone formation while improving the mechanical properties and osteogenic potential of the composite scaffold [16,17]. Other bioactive components have been incorporated into scaffolds for bone regeneration. These include bioactive glass (BG), amorphous calcium phosphate (ACP) nanoparticles, and β-tricalcium phosphate (β-TCP). Previous studies have shown that BG supports osteoblast activity and is essential for cell adhesion, growth, differentiation, and new bone formation. Furthermore, it has been demonstrated that BG degradation can activate gene expression and stimulate the production of growth factors, without inducing inflammation, immune responses, or toxicity *in vivo* [18]. ACP exhibits excellent mechanical properties and enhances the biocompatibility of materials, thereby promoting cell attachment and proliferation. It has also been shown to improve osteoblast adhesion and proliferation [19]. Similarly, β-TCP is widely used in bone regeneration due to its favorable biocompatibility, mechanical strength, and additional biological properties [20,21]. To characterize mechanical and thermal properties, a molecular dynamic simulation method can be applied in order to evaluate the interaction between atoms and molecules of the different components of bio-scaffolds [22].

Platelet lysate (PL), derived from activated platelets, contains a concentrated cocktail of growth factors including platelet-derived growth factor (PDGF), transforming growth factor-β (TGF-β), vascular endothelial growth factor (VEGF), and insulin-like growth factor (IGF), among others [23,24]. These factors play crucial roles in bone healing by promoting angiogenesis, stimulating mesenchymal stem cell recruitment and differentiation, and enhancing osteoblast activity. The incorporation of PL into tissue engineering scaffolds offers the advantage of providing multiple synergistic biological signals that can accelerate bone regeneration processes [25,26].

Hematopoietic stem cells (HSCs) are adult stem cells capable of both self-renewal and differentiation into various blood cell lineages involved in essential biological functions, including the maintenance of homeostasis, immune regulation, and responses to pathogens and inflammation. Moreover, HSCs exhibit a degree of plasticity, allowing them to differentiate into non-hematopoietic cell types such as adipocytes, cardiomyocytes, endothelial cells, fibroblasts/myofibroblasts, hepatocytes, pancreatic cells, and also osteochondrocytes [27,28]. These cells can be isolated from peripheral blood using minimally invasive procedures, eliminating the need for bone marrow aspiration or adipose tissue harvest. When combined with appropriate scaffolds and growth factors, HSCs have demonstrated significant potential for bone regeneration applications [27,28].

This work aims to provide a comprehensive tissue engineering approach that addresses the multifaceted requirements for successful bone regeneration. The integration of silk fibroin scaffolds functionalized with hydroxyapatite and loaded with PL leverages the structural properties of silk fibroin, the osteoconductivity of hydroxyapatite, and the bioactive signaling molecules present in PL to create a biomimetic environment that can effectively support and guide bone tissue formation. Although the literature provides evidence about the osteoinductivity potential of HA-based scaffolds, the novel approach proposed in this study for bone tissue regeneration has not been explored yet. Scaffolds composed of SF, alone and in combination with HA, functionalized with PL have been investigated in this study to assess both the osteoinductive potential of the individual components and the synergistic effect arising from their combination. In addition, understanding the biological response of HSCs to these functionalized scaffolds is essential for optimizing their clinical translation and addressing the significant unmet need in treating non-union fractures and large bone defects.

## 2. Materials and Methods

### 2.1. Preparation of Silk Fibroin Scaffolds

Silk fibroin scaffolds, kindly provided by Caresilk S.r.l.s. (Lecce, Italy), were fabricated by the freeze-drying method through a proprietary process aimed at obtaining a structure suitable for bone tissue regeneration. A methanol post-treatment was applied in the presence of hydroxyapatite (HA) particles (Sigma-Aldrich, St. Louis, MO, USA, 900203, CAS: 1306-06-5); methanol induces the transition from α-helix to β-sheet silk fibroin, while HA particles are physically entrapped within the scaffold structure. Specifically, the scaffolds were incubated under agitation at room temperature for 1 h in a 1% (*w*/*v*) HA suspension in methanol. Both untreated SF scaffolds and HA-impregnated silk fibroin scaffolds (SF-HAs) were subsequently evaluated for their biocompatibility and osteoinductive potential.

### 2.2. Hemopoietic Stem Cells

HSCs from peripheral blood were collected from both healthy donors and patients after obtaining informed consent. Collection was performed via apheresis, followed by cell separation using a density gradient. The harvested cells were subsequently processed and cryopreserved in liquid nitrogen at −196 °C. Specifically, flow cytometry analysis was performed to quantify CD34+-positive cells and the number of these, generally, varies in the range from 400 to 500 CD34+ cells per µL.

For each stored aliquot, a corresponding 1 mL vial was prepared for cell viability analysis. When the primary aliquot is reinfused into the patient, the 1 mL vial is typically discarded. However, as this vial contains a small number of stem cells, it can be used for scientific purposes.

### 2.3. Platelet Growth Factor Collection

Platelets were obtained from single donors via an apheresis procedure using the AMICUS system (Fresenius-Kabi, Modena, Italy). All apheresis collections included online leukoreduction through the R6R2301 kit (Fresenius-Kabi, Modena, Italy). The apheresis was conducted to obtain platelet counts of 1 ± 20% × 10^9^/L, in accordance with national standard guidelines. To produce PL, 4.4 mL of calcium chloride (CaCl_2_) was added per 100 mL of the apheresis-derived platelet product. The mixture was then incubated in a thermostatic water bath at 37 °C for 60 min with continuous stirring. After confirming the formation of a platelet clot, the product was divided into 20 aliquots of 10 mL each, using PRPS 10 bags (Biomed, Modena, Italy). All procedures were carried out under a laminar flow hood. At the end of the process, a small aliquot was used to assess the pH (expected range: 6.4–7.4) and to perform sterility testing through microbiological culture [29].

### 2.4. Biocompatibility Evaluation of Silk Fibroin-Based Scaffolds and Platelet Growth Factors

The biocompatibility of silk fibroin-based scaffolds and platelet growth factors was assessed using primary HSCs. Specifically, the materials were tested both individually and in combination, forming the following experimental groups: (1) SF scaffold, (2) SF-HA scaffold, (3) PGF, (4) SF-PGF, (5) SF-HA-PGF. Tissue culture polystyrene (TCPS) was used as the control (6).

Cell expansion was carried out in Dulbecco’s Modified Eagle Medium (DMEM; Sigma Aldrich) supplemented with 20% fetal bovine serum (FBS), 1% antibiotic solution (100 U/mL penicillin, 100 µg/mL streptomycin), and 2 mM L-glutamine [30]. Cultures were maintained in a humidified incubator (Heracell, Thermo Scientific, Waltham, MA, USA) at 37 °C in an atmosphere of 5% CO_2_. The culture medium and PGF were refreshed every three days. After expansion, the HSCs were seeded in T24 well flasks with a concentration of 1 × 10^4^ cells per well for the SF, SF-HA, SF-PGF, and SF-HA-PGF experimental groups and with a concentration of 5 × 10^3^ for the PGF and TCPS groups.

#### 2.4.1. MTT Assay

The MTT assay [3-(4,5-dimethylthiazol-2-yl)-2,5-diphenyltetrazolium bromide; Sigma Aldrich] was utilized to evaluate the viability of HSCs cultured in contact with the SF, SF-HA, PGF, SF-PGF, and SF-HA-PGF experimental groups. TCPS was used as the control condition. For each group, the assay was carried out in triplicate after 3, 5, and 7 days of culture. A stock MTT solution (5 mg/mL in phosphate-buffered saline—PBS) was added to fresh medium in each well to achieve a final concentration of 0.5 mg/mL. Following a 3 h incubation at 37 °C, the formed formazan crystals were solubilized with Dimethyl Sulfoxide (DMSO). Absorbance readings were taken at 540 nm using a V-1200 spectrophotometer (Avantor, Inc., VWR, Radnor Township, PA, USA) [31,32].

#### 2.4.2. Live/Dead Assay

The biocompatibility of the tested materials was further assessed through a Live/Dead assay conducted on HSCs cultured in contact with SF, SF-HA, PGF, SF-PGF, SF-HA-PGF, and TCPS used as the control. The assay was performed after 7 days of cell culture. Cells grown directly on glass coverslips were incubated at 37 °C for 15 min with a staining solution composed of 2 μmol/L calcein-AM (acetoxymethyl ester of calcein) and 2 μmol/L propidium iodide in PBS used to stain live and dead cells, respectively. Live and dead cells were subsequently visualized using a fluorescence microscope (Axio Vert A1, Zeiss, Oberkochen, Germany) at 20× magnification, and the images were analyzed using AxioVision software (Zeiss ZEN 3.11) [33,34].

#### 2.4.3. Cytoskeleton Structure Analysis

The cytoskeletal organization of HSCs cultured on SF, SF-HA, PGF, SF-PGF, SF-HA-PGF, and TCPS as the control, was evaluated after 7 days of culture. Cells were fixed with 4% paraformaldehyde for 20 min at room temperature, then permeabilized using a 0.5% Triton X-100 solution for 10 min, followed by PBS washing. Actin filaments were stained with tetramethylrhodamine isothiocyanate (TRITC)-conjugated phalloidin (Sigma Aldrich), while cell nuclei were stained with 0.5 mg/mL 4′,6-diamidino-2-phenylindole (DAPI; Invitrogen). Fluorescence imaging was performed at 20× magnification using an Axio Vert A1 microscope (Zeiss), and image analysis was carried out with AxioVision software (Zeiss ZEN 3.11) [35].

### 2.5. Osteoinductivity Propriety Evaluation of Silk Fibroin-Based Scaffolds and Platelet Growth Factors

The osteoinductivity of silk fibroin-based scaffolds and PGFs was assessed through Alizarin Red staining. The assay allows for the evaluation of mineral matrix deposition, which is used as an osteogenic differentiation marker. HSCs were cultured in basal medium under the following experimental conditions: (1) SF, (2) SF-HA, (3) PGF, (4) SF-PGF, (5) SF-HA-PGF, (6) TCPS, as control. Cells were maintained for up to 21 days and the Alizarin Red staining was performed on day 14 and 21. At the expected time points, HSCs cultured in the different experimental conditions were fixed with 4% neutral buffered formalin and stained with a 40 mM Alizarin Red solution (Sigma-Aldrich) at pH 4.2 to evaluate the mineral matrix depositions. After the excess dye was eliminated, the images were acquired using a standard light microscope (Axio Vert A1 microscope; Zeiss) equipped with a digital camera. Subsequently, the bound dye was solubilized using a solution of 20% methanol and 10% acetic acid in water (Sigma-Aldrich). The quantification of the dissolved staining was performed in triplicate using a V-1200 spectrophotometer (Avantor, Inc., VWR) with a wavelength of 450 nm [35].

### 2.6. Statistical Analysis

Statistical experimental analyses were carried out using GraphPad Prism 8.0.1 software. Two-way ANOVA and multiple comparison tests were used to analyze statistics of the MTT assay results, whereas one-way ANOVA and multiple comparison tests were used to analyze the statistics of Alizarin Red staining quantification. *p*-value < 0.05 was considered significant [36,37].

## 3. Results

### 3.1. Biocompatibility of Silk Fibroin-Based Scaffolds and Platelet Growth Factors

**Figure 1:**
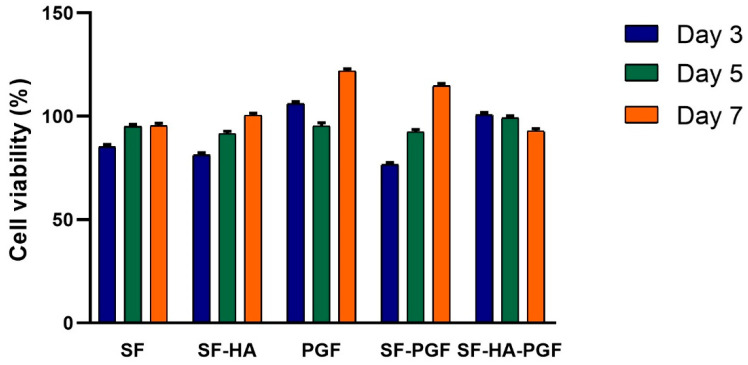
Percentage of cell viability analyzed in HSCs grown in contact with SF, SF-HA, PGF, SF-PGF, SF-HA-PGF, and TCPS at days 3, 5, and 7 and normalized to control group. The percentage of cell viability significantly increased from day 3 to day 7 (*p* < 0.05), as well as from day 5 to day 7 (*p* < 0.05) in the SF, SF-HA, PGF, and SF-PGF experimental groups. In SF-HA-PGF, the percentage of cell viability slightly decreased from day 3 to day 7 without statistical significance (*p* > 0.05). No difference in the percentage of cell viability was found from day 3 to day 5 (*p* > 0.05) and among experimental groups (*p* > 0.05).

The cell viability of HSCs cultured in contact with silk fibroin-based scaffolds and PGFs, either alone or in combination, was evaluated using the MTT assay. Absorbance values obtained for each experimental group were normalized to the control group (TCPS) at the corresponding time point. The results demonstrated an overall increase in cell viability from day 3 to day 7 across the SF, SF-HA, PGF, and SF-PGF experimental groups. Statistically significant differences in cell viability were observed within these groups between day 3 and day 7 (*p* < 0.05), as well as between day 5 and day 7 (*p* < 0.05). The difference in cell viability percentage from day 3 to day 5 was not significant (*p* > 0.05) in all sample’s groups. No statistically significant differences were also detected among the experimental groups at any specific time point (SF vs. SF-HA, SF vs. PGF, SF vs. SF-PGF, SF vs. SF-HA-PGF, SF-HA vs. PGF, SF-HA vs. SF-HA-PGF, PGF vs. SF-PGF, PGF vs. SF-HA-PGF, and SF-PGF vs. SF-HA-PGF; *p* > 0.05). In the SF-HA-PGF group the cell viability slightly decreased but it was not statistically significant (*p* > 0.05), confirming the biocompatibility of the SF-HA-PGF scaffold during the time course. These findings confirm that all tested conditions—(1) SF, (2) SF-HA, (3) PGF, (4) SF-PGF, and (5) SF-HA-PGF—were biocompatible, as they did not induce cytotoxic effects in HSCs (Figure 1).

**Figure 2:**
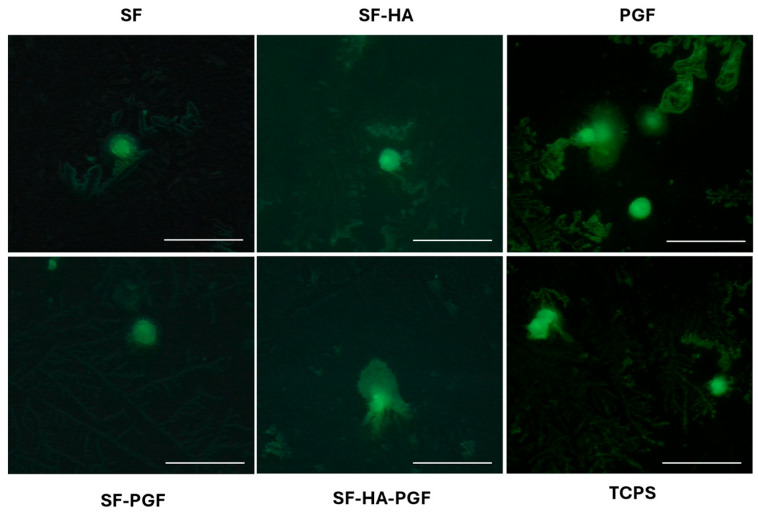
Cell viability analysis of HSCs using Live/Dead assay. Images show live cells grown in contact with SF, SF-HA, PGF, SF-PGF, SF-HA-PGF, and TCPS after 7 days. Dead cells at each time point were not detected. Magnification at 20×; scale bar for each magnification: 50 μm.

The *in vitro* biocompatibility of the (1) SF, (2) SF-HA, (3) PGF, (4) SF-PGF, (5) SF-HA-PGF experimental groups was further assessed using a Live/Dead fluorescence assay on day 7. Live and dead cells were stained with green and red fluorescent dyes, respectively. The fluorescence microscopy images revealed the presence of viable HSC growth in contact with the (1) SF, (2) SF-HA, (3) PGF, (4) SF-PGF, (5) SF-HA-PGF materials, comparable to the cells of the control group (TCPS; Figure 2). Dead cells were not observed, confirming the cytocompatibility of silk fibroin-based scaffolds and platelet growth factors.

**Figure 3:**
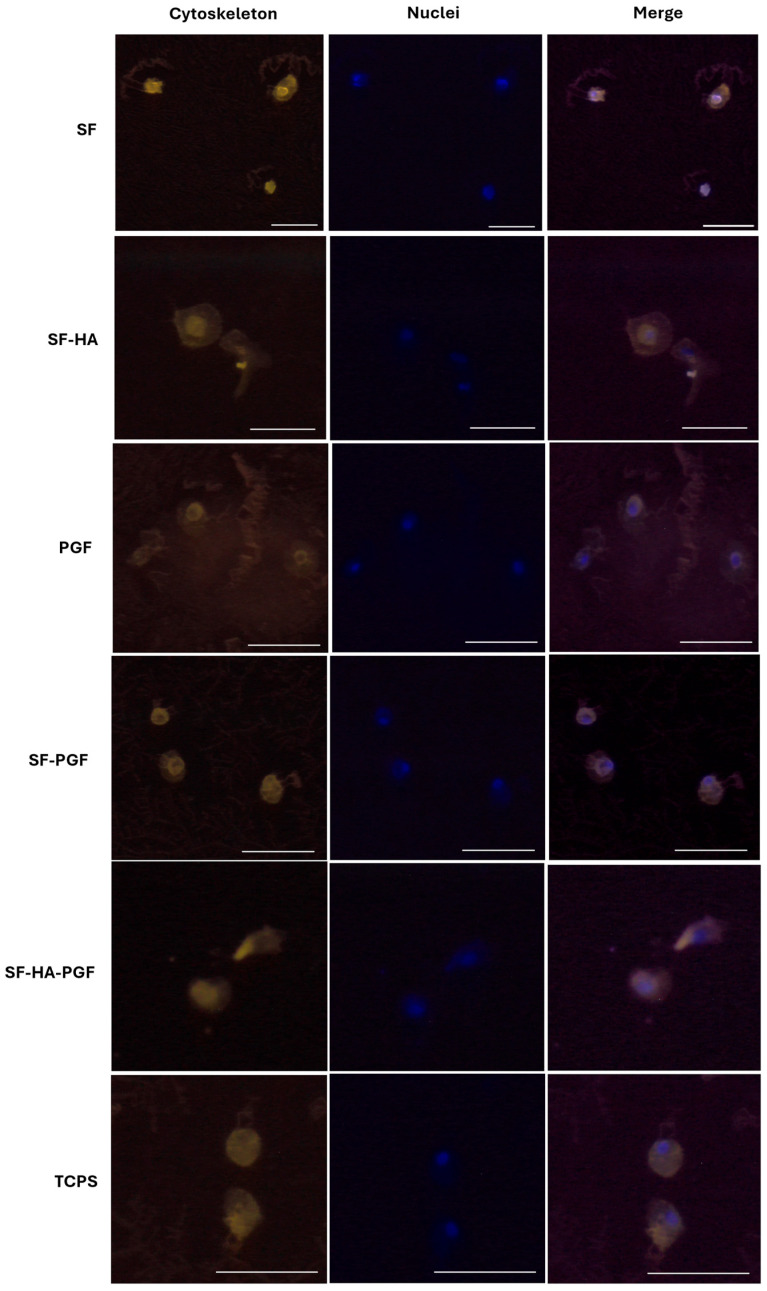
Cytoskeleton analysis of HSCs grown in contact with SF, SF-HA, PGF, SF-PGF, SF-HA-PGF, and TCPS, as control, on day 7. The structure of the cytoskeleton does not show alteration in HSCs grown in contact with SF, SF-HA, PGF, SF-PGF, and SF-HA-PGF compared with the control. Magnification at 20×; scale bar for each magnification: 50 μm.

The cytoskeleton architecture of HSCs growth in contact with (1) SF, (2) SF-HA, (3) PGF, (4) SF-PGF, (5) SF-HA-PGF, as well as on the TCPS control, was assessed via phalloidin-TRITC staining on day 7 (Figure 3). The cytoskeleton structure of HSCs cultured on (1) SF, (2) SF-HA, (3) PGF, (4) SF-PGF, (5) SF-HA-PGF appeared to be well organized, as well as that observed in HSCs growth on the control surface. Fluorescence microscopy images acquired at 20× magnification (Figure 3) showed intact and well-distributed actin filaments, confirming that the silk fibroin-based scaffolds and platelet growth factors, either alone or in combination, supported a normal cytoskeletal architecture and did not compromise cell morphology up to day 7.

### 3.2. Osteoinductivity Propriety of Silk Fibroin-Based Scaffolds and Platelet Growth Factors

Matrix mineralization was evaluated in HSCs cultured on the following substrates for up to 21 days: (1) SF, (2) SF-HA, (3) PGF, (4) SF-PGF, (5) SF-HA-PGF, (6) TCPS as a control. Alizarin Red staining was performed on days 14 and 21 to assess mineral matrix deposition in each experimental group. After staining, cultures were imaged using a bright-field microscope to qualitatively evaluate mineralized areas.

**Figure 4:**
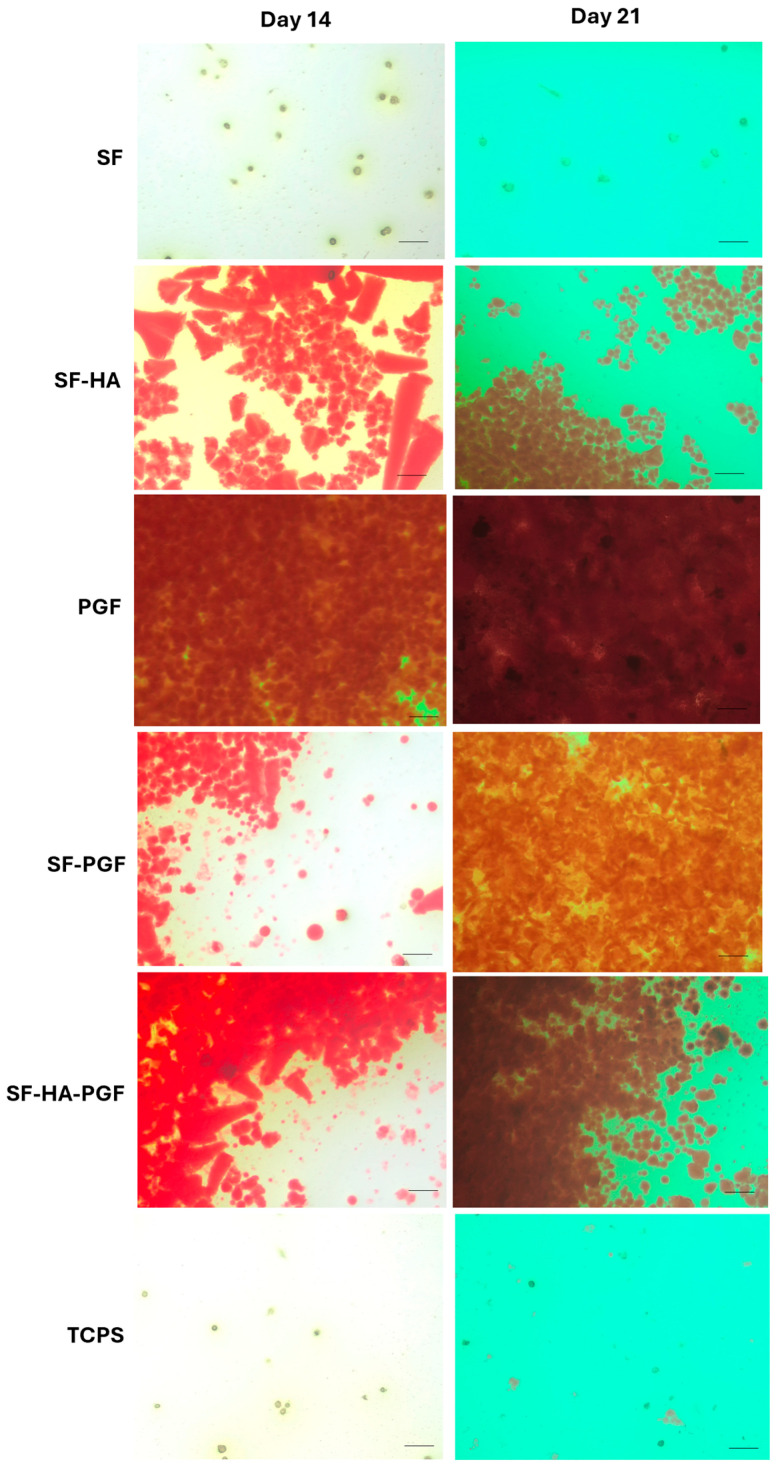
Alizarin Red staining in HSCs grown on SF, SF-HA, PGF, SF-PGF, SF-HA-PGF, and TCPS by day 14 and day 21. Magnification at 10×; scale bar for each magnification: 50 μm.

Digital images on day 14 showed that matrix deposition in the SF group was comparable to the control (TCPS), and this similarity persisted by day 21. In contrast, the SF-HA, SF-PGF, and SF-HA-PGF groups exhibited greater mineral matrix deposition on day 14 compared with both the SF and control groups; this deposition further increased by day 21. Notably, strong mineral matrix formation was observed in the PGF group as early as day 14, which continued through day 21 (Figure 4).

The quantitative analysis of solubilized mineral matrix confirmed the qualitative findings. On day 14, the absorbance in the PGF group was significantly higher than in all other groups (*p* < 0.001), while the absorbance in the SF group remained statistically similar to the control (*p* > 0.05). The SF-HA, SF-PGF, and SF-HA-PGF groups showed significantly increased mineral deposition compared with TCPS (*p* < 0.05), but the difference is not significant among each other (*p* > 0.05).

**Figure 5:**
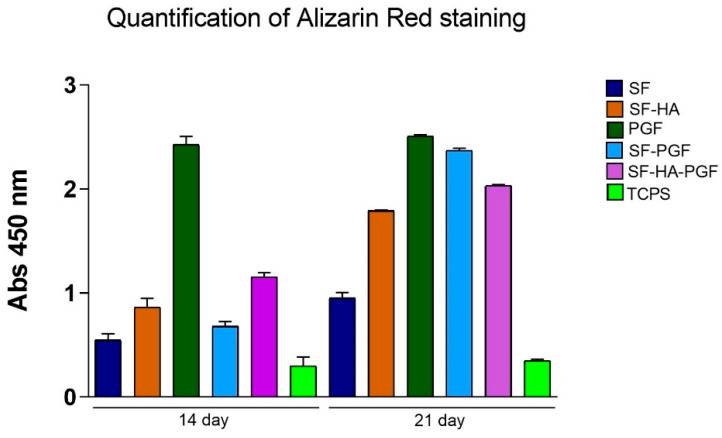
Quantification of Alizarin Red staining through spectrophotometric analysis at 450 nm wavelength measured in HSCs grown on SF, SF-HA, PGF, SF-PGF, SF-HA-PGF, and TCPS.

By day 21, Alizarin Red quantification revealed a significant increase in mineralization in the SF, SF-HA, SF-PGF, and SF-HA-PGF experimental groups compared with day 14 (*p* < 0.001), except for the TCPS and PGF groups for which the quantification was not statistically different (*p* > 0.05) (Figure 5). However, on day 21, the quantification of mineral matrix deposition significantly increased in SF, SF-HA, PGF, SF-PGF, and SF-HA-PGF compared with the control (*p* < 0.001) and in PGF, SF-PGF, and SF-HA-PGF compared with other experimental groups (*p* < 0.05; *p* > 0.001). In addition, on day 21, the quantification of mineral matrix in the PGF group was comparable to the SF-PGF group (*p* > 0.05).

Overall, these results indicate that PGFs alone promote early mineral matrix deposition by HSCs, which remains stable through to day 21. Moreover, silk fibroin-based scaffolds combined with PGFs significantly enhanced mineralization by day 21 compared with scaffolds alone. Interestingly, mineral matrix quantification in the SF-PGF group was comparable to that observed with PGFs alone.

The statistical analysis results at day 14 are as follows: SF vs. TCPS, *p* > 0.05 (*); SF-HA vs. TCPS, *p* < 0.05 (*); SF-PGF vs. TCPS, *p* < 0.05 (*); SF-HA-PGF vs. TCPS, *p* < 0.05 (*); PGF vs. TCPS, *p* < 0.001 (***); PGF vs. SF, *p* < 0.001 (***); PGF vs. SF-HA, *p* < 0.001 (***); PGF vs. SF-PGF, *p* < 0.001 (***); PGF vs. SF-HA-PGF, *p* < 0.001 (***); SF vs. SF-HA, *p* > 0.05 (*); SF vs. SF-PGF, *p* > 0.05 (*); and SF vs. SF-HA-PGF, *p* > 0.05 (*).

The statistical analysis results at day 21 are as follows: SF vs. TCPS, *p* < 0.05 (*); SF-HA vs. TCPS, *p* < 0.001 (***); SF-PGF vs. TCPS, *p* < 0.001 (***); SF-HA-PGF vs. TCPS, *p* < 0.001 (***); PGF vs. TCPS, *p* < 0.001 (***); PGF vs. SF, *p* < 0.001 (***); PGF vs. SF-HA, *p* < 0.001 (***); PGF vs. SF-PGF, *p* > 0.05 (*); PGF vs. SF-HA-PGF, *p* < 0.05 (*); SF vs. SF-HA, *p* < 0.001 (***); SF vs. SF-PGF, *p* < 0.001 (***); and SF vs. SF-HA-PGF, *p* < 0.001 (***).

The statistical analysis across days 14 and 21 also showed the following: SF, SF-HA, SF-PGF, and SF-HA-PGF (*p* < 0.001) (***); PGF and TCPS (*p* > 0.05).

## 4. Discussion

This study demonstrates the efficacious development of functionalized silk fibroin scaffolds that exhibit both excellent biocompatibility and enhanced osteoinductive properties when combined with both hydroxyapatite and platelet growth factors or with platelet growth factors alone. These findings contribute to the growing body of evidence supporting biomimetic approaches in bone tissue engineering. The biocompatibility results confirm that all tested formulations support cell viability and maintain a normal cellular architecture. The consistent increase in cell viability from day 3 to day 7 across all experimental groups indicates that neither the silk fibroin matrix nor the incorporated bioactive components induce cytotoxic effects. This finding aligns with previous studies reporting the excellent biocompatibility of silk fibroin-based materials. Previous studies also report detailed physicochemical and mechanical characterizations of the same base SF scaffolds, including SEM/EDX morphology, tensile/suture strength under hydrated conditions, and swelling/wettability and antibacterial performances, evidencing the high potential of this prototype for the suggested application. In particular, SF scaffolds have exhibited an elongation of 37.41%, a tensile strength of 0.01 MPa, and a swelling ratio of 1200%. Interestingly, a porosity of ~300 μm was obtained, falling within the range generally considered optimal for cell adhesion, proliferation, and extracellular matrix deposition, thus supporting all the biological interactions [13,31].

The preservation of a normal cytoskeletal architecture, as demonstrated by phalloidin staining, further supports the cell-friendly nature of these materials and suggests that the functionalization process does not compromise the scaffold’s ability to support cellular adhesion and spreading.

The use of blood-derived stem cells in this study represents a clinically relevant approach, as these cells can be harvested through minimally invasive procedures compared with bone marrow aspiration. The consistent biocompatibility observed across all experimental groups suggests that these scaffolds could serve as suitable platforms for autologous cell-based therapies. The most significant finding of this study is the enhanced osteoinductive capacity observed when silk fibroin scaffolds are functionalized with bioactive components. The early and sustained mineral matrix deposition in the PGF group confirms the potent osteogenic signaling provided by platelet growth factors. This observation is consistent with the established role of platelets in bone healing, where growth factors such as PDGF, TGF-β, and VEGF orchestrate the complex cascade of bone regeneration [38,39]. TGF-β and their family members, including bone morphogenetic proteins and activins, play an important role in the differentiation of HSCs [40]. TGF-β is a key regulator of osteogenic differentiation, while PDGF acts as both a chemoattractant and a mitogen for osteogenic mesenchymal cells. Notably, PDGF enhances the TGF-β-induced osteogenic differentiation of stem cells through synergistic interactions involving MEK- and PI3K/Akt-mediated signaling pathways [41].

The incorporation of hydroxyapatite into silk fibroin scaffolds (SF-HAs) resulted in enhanced mineralization compared with pure silk fibroin, supporting previous reports that HA serves as a nucleation site for calcium phosphate precipitation and promotes osteoblastic differentiation [16]. The compatibility between the degradation profile of SF–HA scaffolds and the timeline of bone regeneration is a key determinant of their clinical performance. *In vivo* studies have shown that SF scaffolds typically complete degradation within ~2–6 months, a period that can be tuned by modulating β-sheet content, crosslinking, porosity, and processing methods [42,43]. This degradation window overlaps with the hard callus formation phase (≈4–8 weeks) and extends into the onset of the remodeling phase (≥8–12 weeks), thereby ensuring mechanical support during the early stages of osteogenesis and gradual replacement by newly formed bone thereafter [44]. HA contributes long-term osteoconductivity; in particular nano-HA improves integration and mineral metabolism without compromising osteoconductive guidance. SF–HA composites provide a degradation timescale of months, which is well aligned with the biological sequence of callus consolidation and bone remodeling, and thus minimizes the risk of temporal mismatch [45].

However, the most promising results were observed with the combined approach of SF-PGF and SF-HA-PGF, demonstrating the osteoconductive properties of the bioactive signaling molecules present in PL and HA. The osteoinductive results obtained with the scaffolds investigated in this study can be compared to those of commercially available scaffolds clinically used for bone regeneration. For instance, composite materials based on hydroxyapatite and collagen for application in bone regeneration exhibited Alizarin Red values (indicative of osteoinductive capacity and calcium phosphate deposition) lower than those observed with the scaffolds analyzed in the present work. This evidence indicated the strong osteinductive property of the functionalized SF-based scaffold investigated herein [46].

Interestingly, the SF-PGF combination achieved mineralization levels comparable to PGF alone, suggesting the capability of silk fibroin scaffolds to provide an efficient delivery platform.

Although *in vivo* tests are necessary to assess the optimal duration of implantation and subsequent bone tissue formation in clinical application, these findings have addressed important implications for clinical translation, demonstrating the potential of the scaffold as both a structural support and a delivery vehicle for bioactive molecules.

The biomimetic strategy employed in this study addresses multiple aspects of the bone healing process simultaneously. The silk fibroin matrix provides mechanical support and guidance for tissue formation, hydroxyapatite mimics the inorganic component of natural bone, and platelet growth factors replicate the biological signaling during natural bone healing. This multi-component approach may overcome the limitations of current treatment modalities. The enhanced mineralization observed at day 21 suggests that these scaffolds could accelerate bone formation in clinical applications. Given that non-union fractures are defined by a lack of healing progression over 6–8 months, any intervention that can accelerate the initial phases of bone formation could significantly improve patient outcomes and reduce healthcare costs.

## 5. Conclusions

This study investigated a biomimetic strategy for bone tissue engineering based on silk fibroin (SF) scaffolds combined with hydroxyapatite (HA), enriched with platelet-derived growth factors (PGFs) and seeded with hematopoietic stem cells (HSCs). SF-based scaffolds demonstrated good cytocompatibility, maintaining high cell viability and preserved cytoskeletal organization. Functionally, PGFs alone triggered early matrix deposition at day 14, while the presence of SF, with or without HA, sustained and enhanced mineralization at day 21, with the SF-PGF and SF-HA-PGF groups showing a high increase compared with controls. These findings highlight the osteoinductive potential of the functionalized scaffolds and suggest the translational relevance of integrating a natural protein scaffold to recreate a biomimetic environment for bone regeneration, with potential applications in clinical contexts such as delayed union or non-union fractures. Moreover, the use of blood-derived stem cells further strengthens the clinical relevance of this approach, offering a minimally invasive cell source that could facilitate patient-specific applications. Future studies will couple mineralization assays with osteogenic gene/protein expression, while *in vivo* tests will be necessary for the validation and assessment of the scaffold integration and the regenerative performance in clinical practice.

## Data Availability

The original contributions presented in this study are included in the article. Further inquiries can be directed to the corresponding author.
